# Compound Hare-Lip

**Published:** 1879-04

**Authors:** 


### ARTICLE V.

### *Compound Hare-Lip.*

**Proceedings of the New York Academy of Medicine.**

Dr. James L. Little presented three cases of compound hare-lip with cleft palate. The patients were three brothers, aged 22, 18, and 9 years. The father was born in England, the mother was a native of the United States. No deformity could be traced upon either side. The family consisted of nine children, who were born as follows: First, a boy, with compound hare-lip and cleft palate. Second, a girl, without deformity. Third, a boy, with compound hare-lip and cleft palate. Fourth and fifth, two girls without deformity. Sixth, a boy, with compound hare-lip and cleft palate. Seventh and eighth, two girls without deformity. Ninth, a boy, who lived only a few hours, but was born with compound hare-lip and cleft palate. Four boys and five girls; all the boys born with deformity, and all the girls without deformity.

The only external deformity in the family, aside from the hare-lip, was absence of the ring-finger, and a peculiar twisting of the little finger, on the right hand of John Bocock, which gave it somewhat the appearance of a thumb. The index and middle fingers were also larger and longer than corresponding fingers upon the left hand.

On Wm. Bocock, æt. 22 years, four operations were performed. His lip before the operation had a wide cleft, the intermaxillary bone projected and held one incisor tooth, and there was fissure of the hard palate. The first operation was to close the fissure in the hard palate, and was only partially successful; the soft palate separated entirely, and the covering for the fissure in the hard palate almost entirely.

The second operation consisted in removing the projecting portion of the intermaxillary bone, and forming a septum for the nose with the integument by which it was covered. The operation was successful. Subsequently, an operation was performed upon the double hare-lip. That operation was followed by an attack of erysipelas, and was only partially successful.

Lastly, what is known as Nelaton's operation was performed, and the result was very satisfactory.

On John Bocock, æt. 9 years, two operations were performed. The intermaxillary bone projected and carried two incisor teeth. A septum was formed for the nose in the same manner as in the first case, and the operation was successful. Subsequently an operation was performed upon the double hare-lip, and the result was very satisfactory.

Chas. Bocock, æt. 18 years, had a very wide cleft in the mouth, but the projection of the intermaxillary bone was not so prominent as in either of the other cases. Dr. Little proposed to operate after the same general plan by which he had been guided in his operations upon the other patients.

Dr. Post exhibited a *daguerreotype* of a patient thirteen years old, whom he had operated for the correction of a deformity that equalled the worst features of any case which Dr. Little had presented. The result was such that no indentation was left in the margin of the lip. He thought it very important to make the incisions so that when the edges were brought together there would be considerable pouting at the junction on the margin of the lip. There was no danger of bringing too much material to that point.

Again, operations performed upon adults or half-grown children were usually followed by better results than in small children. The parts could be brought into more perfect apposition, etc., still, it was not desirable to allow children to grow up with such a deformity. In cases in which there was fissure in the bony structure, early closure of the fissure in the lip exercised a certain amount of pressure, and diminished the breadih of the cleft in the hard palate.

Dr. Wm. T. White referred to a family in which three of the children were born with hare-lip and fissure of the palate. All died within a few days after birth.

Dr. Post referred to a family reported by Dr. Buck, in which the mother and five or six children had hare-lip.

Dr. Garrish referred to four families in which there were three children in each family who had hare-lip, either compound or simple.

Dr. Weiner referred to a family in which two children born of the first wife had compound hare lip, but in none of the children born of the second wife did the deformity appear.

He thought it was next to impossible to secure such perfect coaption of the parts at the first operation as could entirely correct the deformity. He had never seen a case in young children in which it was not necessary, after the child reached ten or twelve years of age, to operate to remedy the indentation in the margin of the lip.

With reference to closing the fissure in the hard palate, he thought an operation should be avoided, because the dentists had succeeded so well in making an artificial roof of the mouth, and at the same time the operation was quite commonly unsuccessful.

Dr. Little thought if the operation could be performed it was usually successful, and believed it to be bad advice to send such patients to the dentists. There were comparatively few patients who could bear the expense of the artificial appliance. In order to secure the best results so far as indentation in the margin of the lip was concerned, he thought it very important to secure perfect coaptation at the *upper* edge of the vermilion border.

Dr. Weiner referred to a case in which an opening in the hard palate about three-fourths of an inch in diameter was successfully closed by a button made of gutta-percha. The girl had learned to mould one herself, and made a new button as often as the old one became hard and gave her any discomfort. An artificial appliance was thus afforded which answered all practical purposes, and the expense was a mere trifle.

Dr. Post thought it well confirmed that a properly formed artificial palate was more useful than a united velum, and was preferable if the patient could the expense.—*Medical Record.*

---

